# Circular RNA ERBIN Promotes Proliferation of Hepatocellular Carcinoma *via* the miR-1263/CDK6 Axis

**DOI:** 10.3389/fonc.2022.878513

**Published:** 2022-04-21

**Authors:** Shikun Yang, Fei Yu, Yang Ji, Yanjun Shen, Hao Lu, Yuan Gao, Feng Zhang, Xuehao Wang, Chuanyong Zhang

**Affiliations:** ^1^ Hepatobiliary Center, The First Affiliated Hospital of Nanjing Medical University, Key Laboratory of Liver Transplantation, Chinese Academy of Medical Sciences, Nanjing, China; ^2^ Department of Hepato−Biliary−Pancreatic Surgery, The Affiliated Changzhou No. 2 People’s Hospital of Nanjing Medical University, Changzhou, China

**Keywords:** HCC, circERBIN, proliferation, miRNA sponge, miR-1263, CDK6

## Abstract

**Objectives:**

Hepatocellular carcinoma (HCC) is the most common primary liver cancer and characterized by high aggressiveness and extremely poor prognosis. Increasing evidence has suggested that circular RNAs (circRNAs), which are highly stable, play crucial roles in the progression of multiple malignancies. However, the roles of circRNAs in HCC remain elusive.

**Materials and Methods:**

The expression patterns of circRNAs in HCC were identified by qRT-PCR. A series of functional experiments both *in vivo* and *in vitro* were used to determine the role of circERBIN in HCC proliferation. Bioinformatics and an RNA pulldown assay were used to identify potential downstream targets of circERBIN.

**Results:**

The expression of circERBIN was upregulated in HCC cell lines and tissues, which was predictive of a poor prognosis in HCC patients. Elevated circERBIN promoted G1/S transition of HCC cells, thus facilitating the proliferation and tumorigenesis of HCC cells. Mechanistic investigations revealed that circERBIN regulated HCC proliferation by acting as a sponge of miR-1263, which subsequently targeted cyclin dependent kinase 6 and controlled G1/S transition.

**Conclusion:**

Taken together, these results determined that circERBIN functions as an important epigenetic regulator in HCC development, highlighting that circERBIN is a promising target for treatment of HCC.

## Introduction

Hepatocellular carcinoma (HCC) is the sixth most common malignant cancer and the third leading cause of cancer-related death worldwide (1). The overall survival of HCC patients remains extremely poor due to high disease aggressiveness and malignant biological behavior. Although significant progress has been made in the treatment of HCC, the effects of current strategies, such as targeted molecular therapy, surgical treatment, and immunotherapy, remain largely unsatisfactory (2, 3). Hence, further efforts are needed to elucidate the molecular mechanisms underlying the development and progression of HCC to improve the therapeutic outcomes of HCC patients.

Circular RNAs (circRNAs) are a series of widespread and conserved non-coding RNA that are mostly generated by direct back-splicing of precursor mRNA (4, 5). Accumulating evidence has demonstrated that aberrantly expressed circRNAs are involved in diseases progression, particularly malignant cancers.

With the development of next-generation sequencing of noncoding RNAs, a growing number of circRNAs have been found play important roles in cancer cell proliferation, metastasis, and treatment resistance by functioning as microRNA (miRNA) sponges and RNA-binding proteins (6–8). For instance, our group previously reported that circ_0011385 promoted HCC cell proliferation and was associated with poor clinicopathologic features (9). A recent study showed that hsa_circ_0001492 (circERBIN), originating from exons 2, 3, and 4 of the Erbin gene (ERBB2 inter-acting protein), promoted the proliferation and metastasis of colorectal cancer cells *via* targeting miR-125a-5p-5p/miR-138-5p to subsequently increase the expression of eukaryotic translation initiation factor 4E binding protein 1 and translation of hypoxia induced factor-1 (10). Nevertheless, little is known about the expression patterns and role of circERIN in the carcinogenesis and progression of HCC.

The results of this study showed that upregulation of circERIN was correlated with poor clinicopathologic outcomes of HCC. In addition, CircERIN facilitated proliferation of HCC cells both *in vivo* and *in vitro*. Mechanistically, circERIN regulates the cell cycle by sponging miR-1263 and regulating the expression of cyclin dependent kinase 6 (CDK6). Therefore, circERBIN may be considered as a promising therapeutic target for HCC patients.

## Materials and Methods

### Cell Culture and Tissue Samples

Forty-four samples of HCC and paired adjacent liver tissues were obtained from patients who underwent surgery at the First Affiliated Hospital of Nanjing Medical University (Nanjing, China). All procedures were approved by Ethics Committee of the First Affiliated Hospital of Nanjing Medical University. HCC cell lines and normal hepatic cells were purchased from the Cell Bank of the Chinese Academy of Sciences (Shanghai, China) and cultured in Dulbecco’s modified Eagle’s medium (HyClone Laboratories, Inc., South Logan, UT, USA) supplemented with 10% fetal bovine serum (Gibco, Carlsbad, CA, USA) and 1% penicillin-streptomycin solution (HyClone Laboratories, Inc.).

### Transfection Experiments

Short interfering RNA (si-circERBIN), miR-1263 inhibitors, mimics, and appropriate negative controls (NCs) were designed by TSINGKE Biotechnology Co., Ltd. (Beijing, China). Lipofectamine 3000 transfection reagent (Invitrogen Corporation, Carlsbad, CA, USA) was used for transient transfection. Lentiviruses coding for circERBIN, CDK6, and shCDK6 were constructed by GenePharma Co., Ltd. (Shanghai, China).

### Western Blot Analysis

Proteins were extracted from cells using radioimmunoprecipitation assay buffer containing phenylmethylsulfonyl fluoride. The quality of the protein samples was evaluated using a NanoDrop ND-2000 spectrophotometer (Thermo Fisher Scientific, Waltham, MA, USA). Equal amounts of protein were separated by sodium dodecyl sulfate-polyacrylamide gel electrophoresis and then transferred onto polyvinylidene fluoride membranes (Merck Millipore, Billerica, MA, USA), which were blocked with Quick Block™ Blocking Buffer for Western Blot (Beyotime Institute of Biotechnology, Haimen, China) and incubated with primary antibodies against CDK6 (13331S; Cell Signaling Technology, Inc., Danvers, MA, USA) and glyceraldehyde-3-phosphate dehydrogenase (GAPDH; 5174T; Cell Signaling Technology, Inc.) overnight at 4°C. The next day, the membranes were incubated with corresponding horseradish peroxidase-labelled secondary antibodies at room temperature for 2 h. Afterward, the membranes were visualized using an enhanced chemiluminescence detection system.

### Quantitative Real-Time Polymerase Chain Reaction (qRT-PCR)

Total RNA was extracted and reverse transcribed into complementary DNA with forward and reverse primers targeting circERBIN (TAC CAG CAT CCA TTG CAA AC/TCC TCT TCC CCT CGT AGA CA), miR-1263 (AAC AAG ATG GTA CCC TGG CAT AC/CAG TGC AGG GTC CGA GGT), CDK6 (CAG CAG CGG ACA AAT AAA/CTG GGA GTC CAA TCA CGT), and GAPDH (AAG GTG AAG GTC GGA GTC A/GGA AGA TGG TGA TGG GAT TT). The qRT-PCR cycling conditions included an initial denaturation step at 95°C for 5 min, followed by 95°C for 10 s and 60°C for 30 s, and 40 cycles at 95°C for 15 s, 60°C for 1 min, and 95°C for 15 s.

### Fluorescence *In Situ* Hybridization (FISH)

The FISH assay was conducted using a Ribo FISH kit (Guangzhou RiboBio Co., Ltd., Guangzhou, China) with probes targeting circERBIN, which were designed and synthesized by Guangzhou RiboBio Co., Ltd. All FISH procedures were conducted in accordance with the manufacturer’s protocols.

### Flow Cytometry

Transfected cells (1 × 10^6^) were harvested and stored in 75% ethanol at −20°C overnight. After centrifugation, the cells were stained with DNA staining solution [Multisciences (Lianke) Biotech Co., Ltd, Hangzhou, China]. Analysis was conducted using a BD™ LSR II flow cytometer (BD Biosciences, San Jose, CA, USA).

### Cell Counting Kit-8 (CCK-8), EdU Cell Proliferation, and Colony Formation Assays

The CCK-8, EdU cell proliferation, and colony formation assays were performed as previously reported.

### RNA Pulldown Assay

MiRNAs bound to circERBIN were detected using the Pierce Magnetic RNA-Protein Pull-Down Kit (Thermo Fisher Scientific) in accordance with the manufacturer’s instructions with biotin-labeled probes designed and synthesized by Guangzhou RiboBio Co., Ltd.

### *In Vivo* Tumor Models

Four-week-old nude male mice were purchased from the Animal Core Facility of Nanjing Medical University and assigned to one of two groups. Then, each mouse was subcutaneously injected with 2 × 10^6^ cells. After two weeks, mice with palpable tumors were injected intratumorally with 50 nmol cholesterol-conjugated si-NC or si-circERBIN 3 times a week for 2 weeks. Tumor volumes were measured weekly. After 5 weeks, all mice were sacrificed and the tumor tissues were harvested and weighed.

### Luciferase Reporter Assay

Luciferase reporter plasmids coding for mutant (MUT) and wild-type (WT) circERBIN and CDK6 were constructed by Shanghai GeneChem Co., Ltd. (Shanghai, China). Cells were transfected with the plasmids using Lipofectamine 3000 transfection reagent and activity was measured with the Dual-Luciferase Reporter System Kit (E1910; Promega Corporation, Madison, WI, USA).

### Statistical Analysis

Statistical analyses were performed using GraphPad Prism 9.0 software (GraphPad Software, Inc., San Diego, CA, USA) and IBM SPSS Statistics for Windows, version 24.0. (IBM Corporation, Armonk, NY, USA). Data are expressed as the mean ± standard deviation. The Student’s *t*-test was used to identify differences between the control and experimental groups. The log-rank test was used to assess the survival data, which are presented as Kaplan–Meier survival curves. Correlations between groups were analyzed using Pearson’s test. The chi-squared test was performed to analyze the association between the expression levels of target genes and clinicopathological parameters.

## Results

### circERBIN Is Upregulated in HCC Cells and Tissues

**Figure 1:**
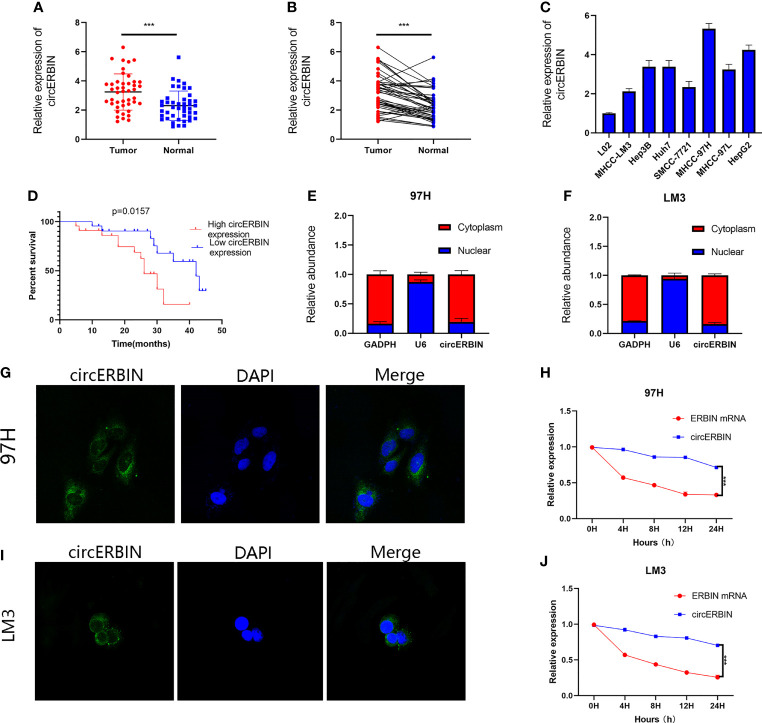
CircERBIN is upregulated in HCC cells and tissues. **(A, B)** The relative expression of circERBIN was determined in 44 pairs of HCC and para-cancer tissues by qRT-PCR. **(C)** The relative expression of circERBIN was examined in HCC cell lines by qRT-PCR. **(D)** Kaplan–Meier survival curve analysis showed that high expression of circERBIN was associated with shorter overall survival of HCC patients. **(E, F)** qRT-PCR analysis of circERBIN abundance in the cytoplasmic and nuclear fractions of 97H and LM3 cells. GAPDH and U6 were used as positive controls in the cytoplasm and nucleus, respectively. **(G, H)** FISH was used to determine the localization of circERBIN in HCC cells. **(I, J)** Time-course qRT-PCR analyses of the relative abundance of circERBIN and linear ERBIN in LM3 and 97H cells treated with actinomycin D (10 μg/mL). (***p < 0.001).

The qRT-PCR results revealed that circERBIN was significantly upregulated in HCC tissues as compared with para-cancerous normal tissues (**Figures 1A, B**). Among seven HCC cell lines and one normal cell line, circERBIN expression was highest in 97H cells and lowest in LM3 cells (**Figure 1C**). Furthermore, upregulation of circERBIN was associated with lower overall survival of HCC patients (**Figure 1D**). As shown in **Table 1**, circERBIN was significantly correlated with tumor size, Edmondson grade, and TNM stage, but not age, sex, hepatitis B virus infection, and liver cirrhosis. Next, the localization of circERBIN in HCC cells was investigated. The nuclear-cytoplasmic fractionation experiments (**Figures 1E, F**) and FISH assays (**Figures 1G, H**) demonstrated that circERBIN was mainly localized in the cytoplasm. To further evaluate the circular structure of circERBIN, the actinomycin D assay (**Figures 1I, J**) was performed. The results showed that circERBIN was more stable than linear ERBIN.

**Table 1:** Correlation between circERBIN expression and clinicopathological features in HCC tissues (n = 44, χ2 -test).

Variable	circERBIN expression		P-value
	high	low	
	22	22	
Age (year)			0.7609
<60	13	12	
≥60	9	10	
Gender			0.7569
Male	8	13	
Female	14	9	
Tumor size			0.0346*
<5 cm	10	14	
≥5 cm	12	8	
TNM stage			0.0142*
I–II	9	17	
III–IV	13	5	
Liver cirrhosis			0.7628
Yes	12	11	
No	10	11	
AFP (ng/mL)			0.0658
≤200	6	12	
>200	16	10	
HBsAg			0.7505
positive	15	14	
negative	7	8	
Edmondson grade			0.0053**
I–II	4	13	
III–IV	18	9	

*p < 0.05, **p < 0.001.

### circERBIN Promotes the Proliferation and G1/S Transition of HCC Cells

**Figure 2:**
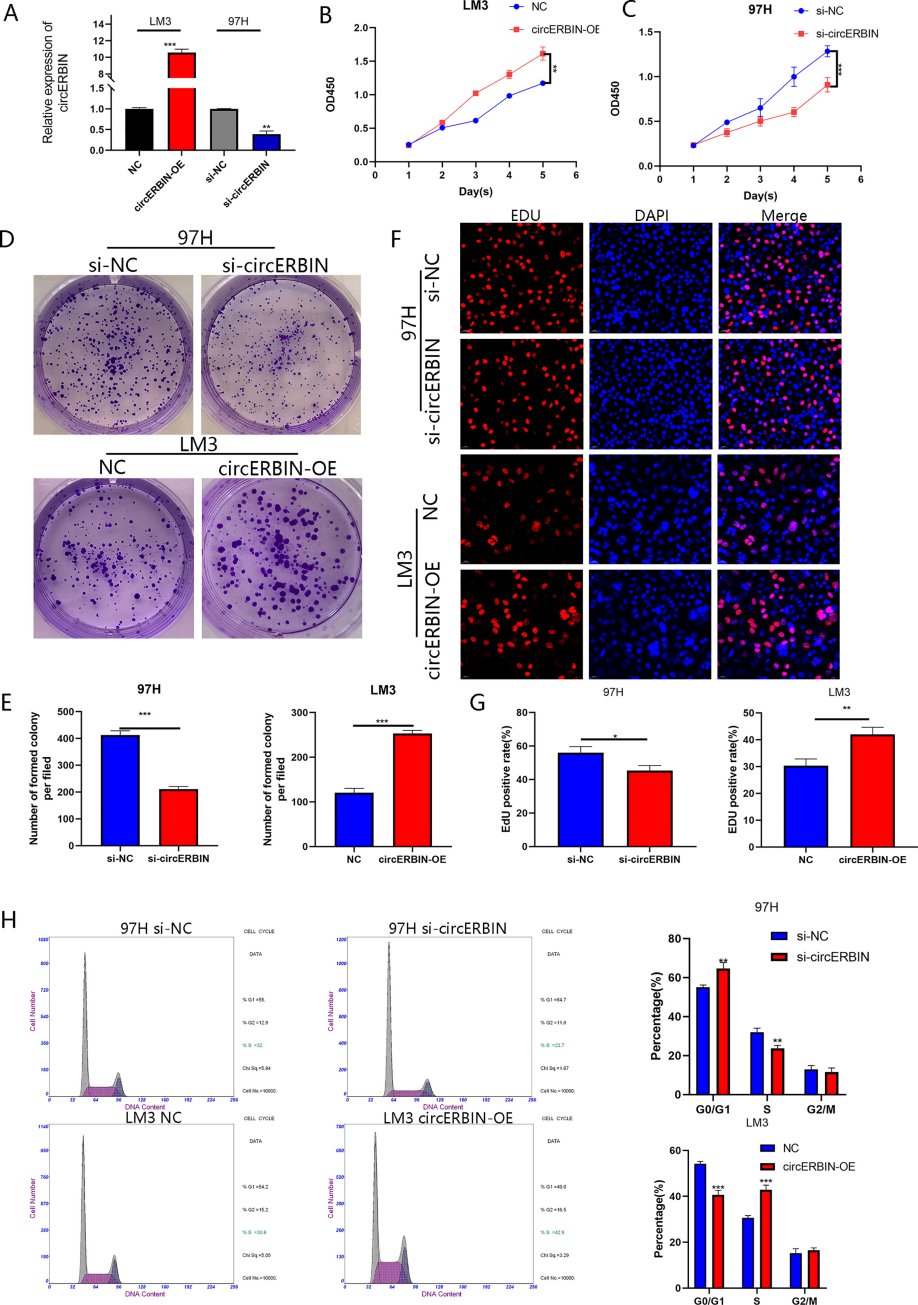
CircERBIN promotes the proliferation and G1/S transition of HCC cells. **(A)** Expression of circERBIN was confirmed by qRT-PCR in HCC cells transfected with NC, circERBIN, si-NC, or si-circERBIN. **(B)** CCK-8 assay of 97H cells after circ-ERBIN knockdown. **(C)** CCK-8 assay of LM3 cells overexpressing circERBIN. **(D, E)** Effects of si-ERBIN and circERBIN-OE on proliferation of HCC cell lines by a colony formation assay. **(F, G)** EdU assays of LM3 and 97H cells treated with circERBIN or si-circERBIN. **(H)** Flow cytometry assays showing that circERBIN can accelerate G1 to S phase transition, while circERBIN knockdown increased the proportion of cells in the G1 phase. (*p < 0.05, **p < 0.01, ***p < 0.001).

To investigate the biological role of circERBIN in HCC, LM3 cells were transfected with plasmids overexpressing circERBIN (**Figure 1A**). In addition, circERBIN expression was silenced in 97H cells by transfection with short interfering (si) RNA (**Figure 2A**). Knockdown of circERBIN significantly impaired the proliferation of 97H cells as compared with the si-NC, as demonstrated by the CCK-8 assay (**Figure 2B**), while overexpression of circERBIN accelerated the proliferation of LM3 cells (**Figure 2C**). The results of the colony formation assay showed that 97H cells transfected with si-circERBIN produced fewer colonies as compared with the control cells (**Figures 2D, E**). In addition, the colony formation rate was higher in LM3 cells overexpressing circERBIN (**Figures 2D, E**). The results of the EdU cell proliferation assay (**Figures 2F, G**) were consistent with those of the colony formation and CCK-8 assays. Besides, flow cytometry was performed to assess the proportions of cells in each stage of the cell cycle. The results revealed that silencing of circERBIN increased the proportion of 97H cells in the G0/G1 phase, while overexpression of circERBIN had an opposite effect in LM3 cells (**Figure 2H**). These results demonstrated that circERBIN was a tumor promotor and accelerated the proliferation of HCC cells *in vitro.*

### circERBIN Promotes the Proliferation of HCC Cells *In Vivo*

**Figure 3:**
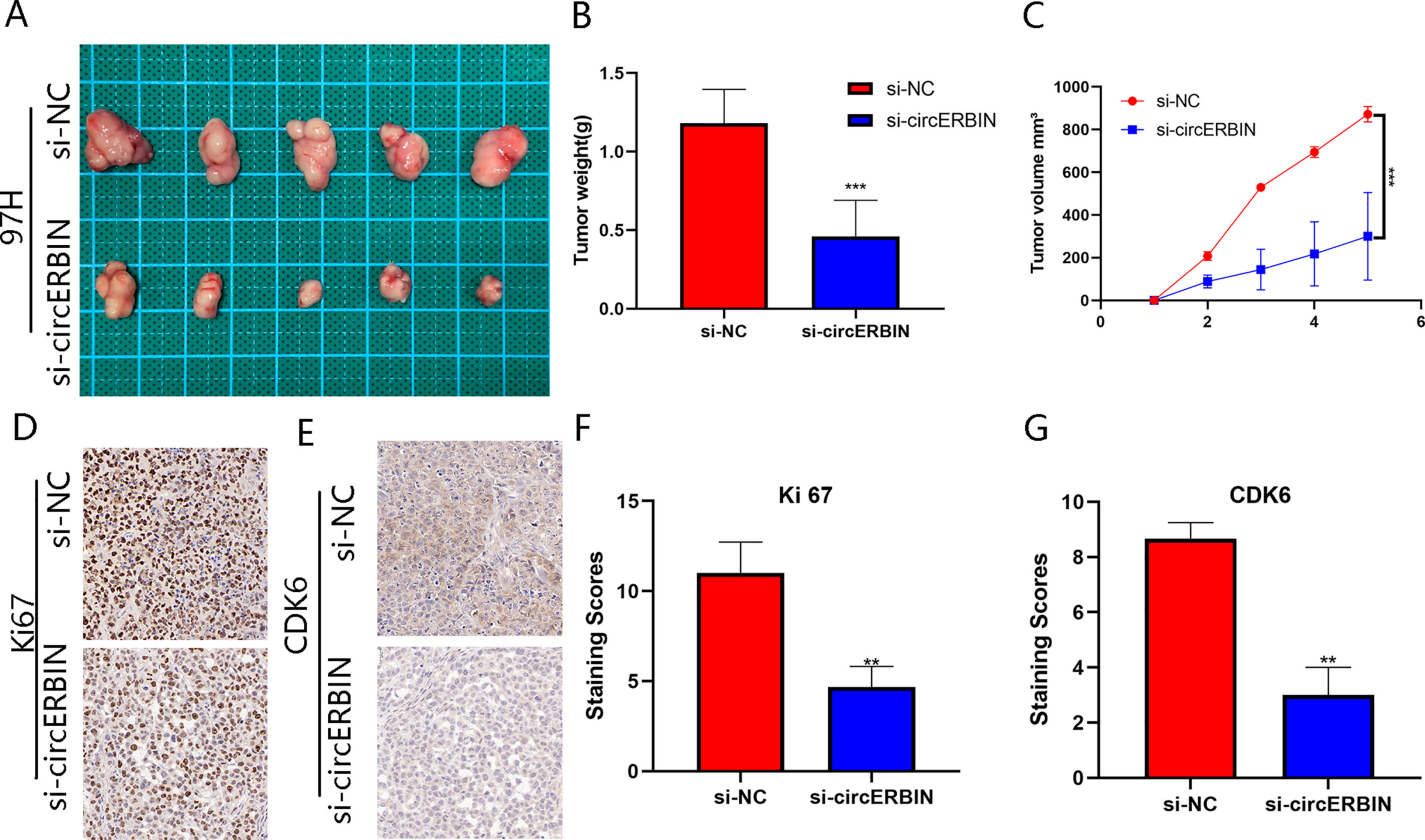
CircERBIN promotes the proliferation of HCC cells *in vivo.* **(A–C)** 97H cells transfected with si-circ-ERBIN were injected into BALB/C nude mice (n = 5/group). Tumor volumes and weights were monitored. **(D, F)** IHC staining of xenograft tumors. The protein levels of Ki67 were analyzed based on IHC staining. **(E, G)** IHC staining of xenograft tumors. The protein levels of CDK6 were analyzed based on IHC staining. The samples were imaged at 400× magnification. (**p < 0.01, ***p < 0.001).

To determine whether circERBIN functions as a tumor promotor *in vivo*, nude mice were subcutaneously injected with HCC cells (**Figure 3A**). Tumor volumes were measured and recorded weekly (**Figure 3C**). After 5 weeks, all mice were sacrificed and tumor tissues were harvested and weighed (**Figure 3B**). Additionally, IHC staining showed a reduction Ki-67 levels in tumor cells after circERBIN knockdown (**Figures 3D, F**). The results revealed that downregulation of circERBIN significantly suppressed tumor growth, thereby further verifying the role of circERBIN in HCC tumorigenesis *in vivo*.

### miR-1263 Is Sponged by circERBIN

**Figure 4:**
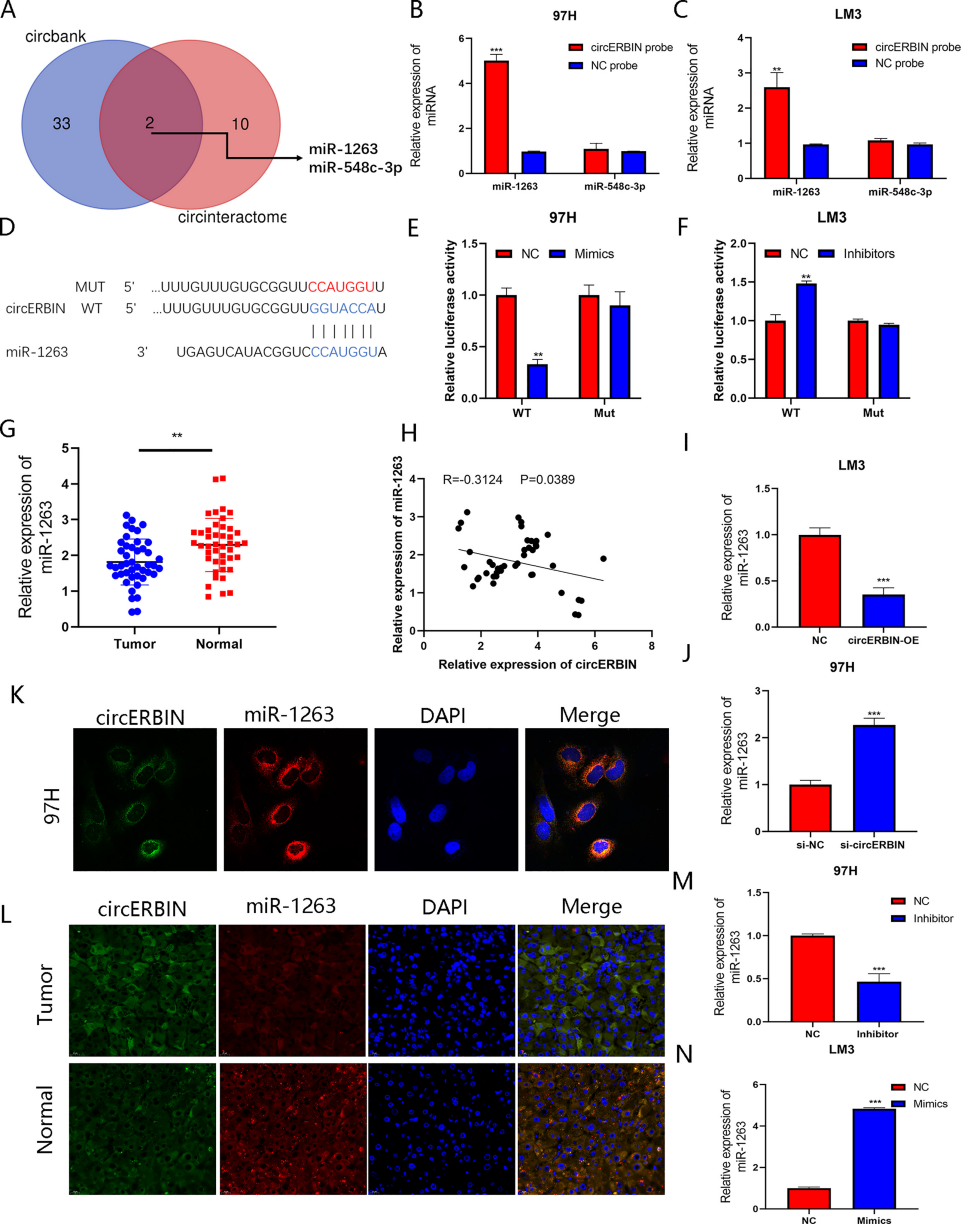
miR-1263 is sponged by circERBIN. **(A)** Schematic illustration exhibiting overlapping of the target miRNAs of circERBIN as predicted by the circBank and CircInteractome database. **(B, C)** The relative abundances of two miRNA candidates in 97H and LM3 lysates with circERBIN or oligonucleotide probes were examined by qRT-PCR. **(D)** Schematic illustration of the sequence of WT and MUT circERBIN at the miR-1263 binding site. **(E, F)** The effects of miR-1263 mimics, an inhibitor, and NC on luciferase activity were detected in HCC cells transfected with luciferase reporter plasmids. **(G)** Relative miR-1263 expression in 44 pairs of fresh frozen HCC tissues and matched normal liver tissues. **(H)** An obvious negative correlation between the levels of circERBIN and miR-1263 in 44 pairs of fresh frozen HCC tissues and matched normal liver tissues as determined by Pearson correlation analysis. **(I, J)** The relative levels of miR-1263 in 97H and LM3 cell lines transfected with si-circERBIN, circERBIN, or NC as detected by qRT-PCR. **(K)** Co-localization of circERBIIN and miR-1263 in HCC cells as detected with the FISH assay. Scale bar, 10 μm. **(L)** FISH results showing co-localization of circERBIIN and miR-1263 in HCC and para-cancerous tissues from patients. Scale bar, 25 μm. **(M, N)** Relative miR-1263 expression in transfected 97H and LM3 cells. (**p < 0.01, ***p < 0.001).

Considering that circRNAs have been reported as sponges of miRNAs and regulate mRNA expression, we hypothesized that circERBIN functions *via* a mechanism dependent on competing endogenous RNA. To determine whether circERBIN can bind to miRNA, possible targets of circERBIN were predicted with the online databases circBank (http://www.circbank.cn/) and CircInteractome (http://circinteractome.nia.nih.gov/) (**Figure 4A**). Two candidate miRNAs (miR1263 and miR-548c-3p) with potential binding sites for circERBIN were identified. The results of a pull-down assay using biotin-labeled probes showed significant fold changes of miR-1263 as compared with a NC (**Figures 4B, C**). To further verify the interaction between circERBIN and miR-1263, luciferase reporter plasmids were constructed containing the full length WT or MUT sequence of circERBIN within the binding sites of miR-1263 (**Figure 4D**). Then, HCC cells were co-transfected with the luciferase plasmids and miR-1263 mimics or inhibitors. The results showed that the miR-1263 mimics significantly decreased the luciferase activity of the WT (**Figure 4E**), but not the MUT, while the miR-1263 inhibitor had an opposite effect (**Figure 4F**). These findings indicate that there could be a direct interaction between circERBIN and miR-1263. Moreover, miR-1263 expression was notably downregulated in HCC tissues (**Figure 4G**), while circERBIN was negatively correlated with miR-1263 (**Figure 4H**). The FISH results indicated that circERBIN and miR-1263 co-localized in the cytoplasm of HCC cells and tissues (**Figures 4K, L**). In contrast to miR-1263, circERBIN levels were increased in tumor tissues. In addition, overexpression of circERBIN inhibited the expression of miR-1263, whereas silencing of circERBIN had a significant opposite effect (**Figures 4I, J**). Taken together, these results suggest that circERBIN may function as a sponge for miR-1263.

### miR-1263 Inhibits the Proliferation of HCC Cells

**Figure 5:**
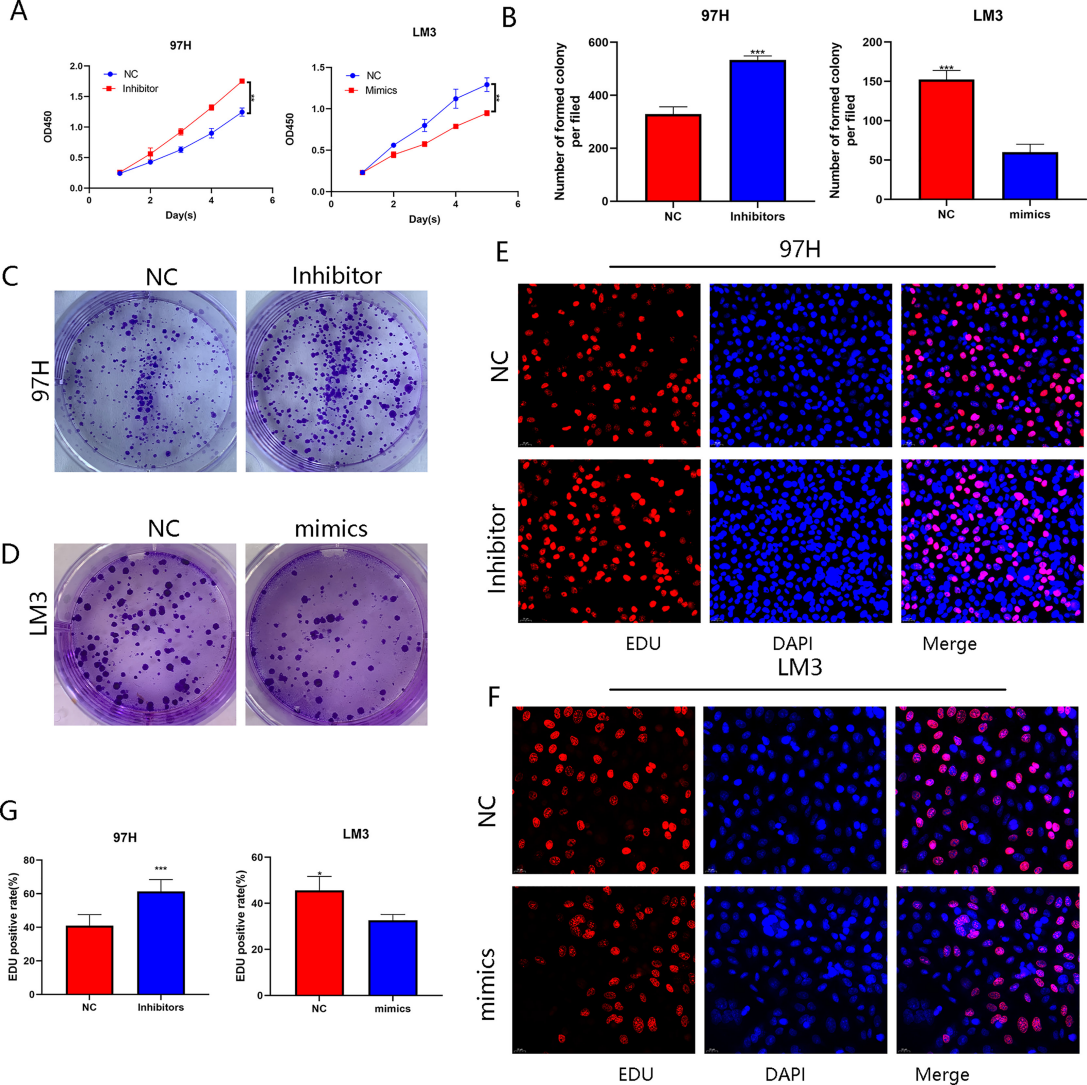
miR-1263 inhibits the proliferation of HCC cells. **(A)** CCK-8 assay of LM3 and 97H cells transfected with mimics or an inhibitor. **(B–D)** Effect of mimics or an inhibitor on the proliferation in HCC cell lines as determined by the colony formation assay. **(E–G)** EDU assay of LM3 and 97H cells treated with mimics or an inhibitor. (*p < 0.05, **p < 0.01, ***p < 0.001).

To further elucidate the effects of miR-1263-mediated regulation of HCC cells, LM3 and 97H cells were transfected with miR-1263 mimics or inhibitors. The transfection efficiency was verified by qRT-PCR (**Figures 4M, N**). The results of the CCK-8 (**Figure 5A**), colony formation (**Figures 5B–D**), and EdU cell proliferation (**Figures 5E–G**) assays confirmed that overexpression of miR-1263 inhibited the proliferation of 97H cells, while silencing miR-1263 remarkably decreased these cellular behaviors.

### CDK6 Is a Direct Target of miR-1263

**Figure 6:**
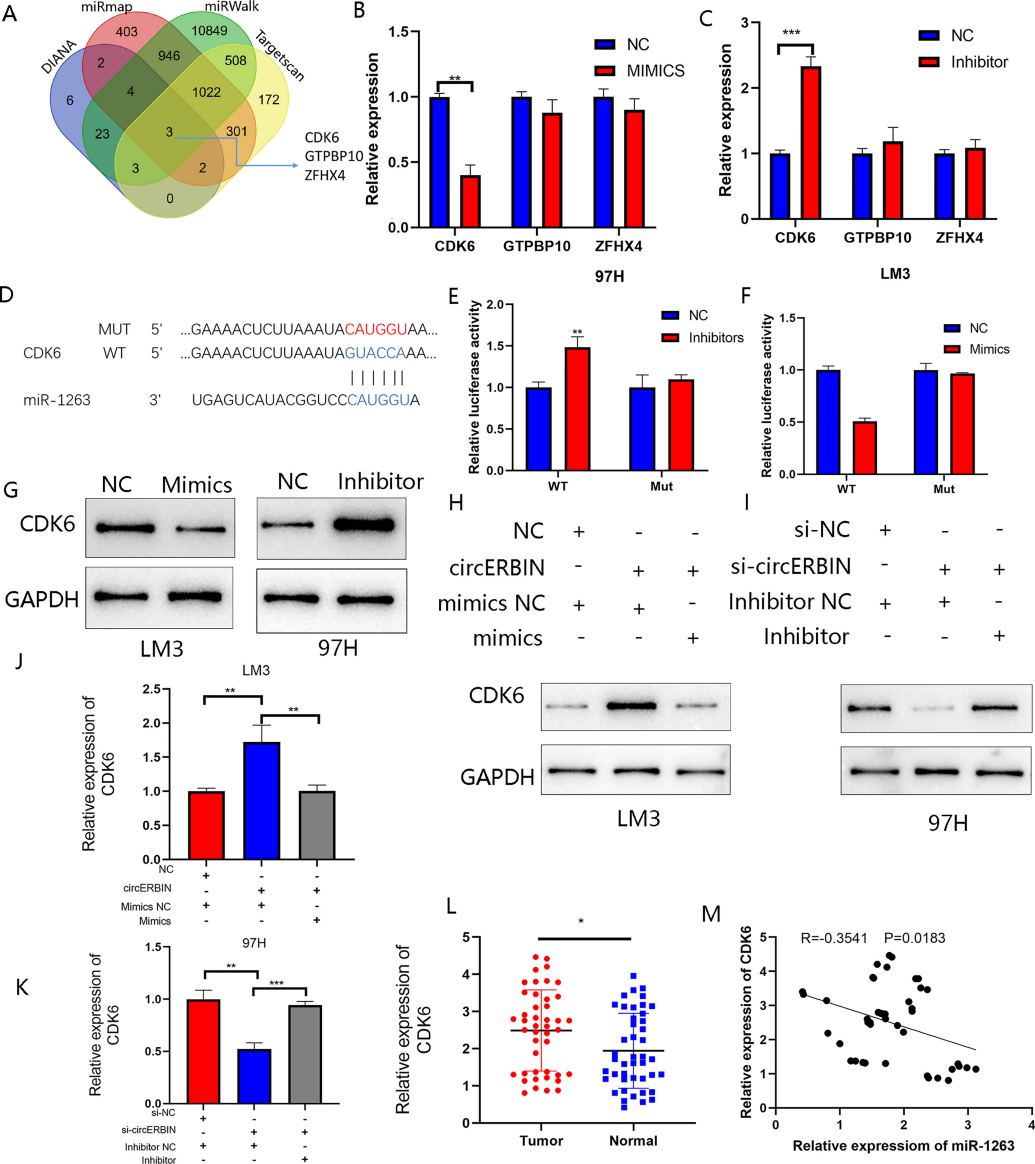
CDK6 is a direct target of miR-1263. **(A)** Schematic illustration exhibiting overlapping of the target mRNAs of miR-1263 predicted by the DIANA, miRmap, miRwalk, and Targetscan databases. **(B, C)** Expression of predicted target genes in LM3 and97H cells transfected with miR-1263 mimics, an inhibitor, and corresponding NC. **(D)** Schematic illustration of the WT and MUT CDK6 sequences at the miR-1263 binding site. **(E, F)** The effects of miR-1263 mimics and inhibitor on the luciferase activities of the WT and MUT CDK6 mRNA 3’-untranslated regions as detected by qRT-PCR. **(G)** The effects of miR-1263 on the protein expression of TDO2 were detected by western blot analysis. **(H, I)** Western blotting showing CDK6 protein expression in transfected 97H and LM3 cells. **(J, K)** QRT-PCR showing CDK6 mRNA expression in transfected 97H and LM3 cells. **(L)** Relative CDK6 expression in 44 pairs of fresh frozen HCC tissues and matched normal liver tissues. **(M)** An obvious negative correlation between the levels of miR-1263 and CDK6 in 44 pairs of fresh frozen HCC tissues and matched normal liver tissues as determined by Pearson correlation analysis. (*p < 0.05, **p < 0.01, ***p < 0.001).

The DIANA (http://diana.imis.athena-innovation.gr/DianaTools/index.php), miRmap (https://mirmap.ezlab.org/), miRwalk (http://mirwalk.umm.uni-heidelberg.de/), and Targetscan (https://www.targetscan.org/vert_80/) bioinformatic tools were used to predict the downstream targets of miR-1263 (**Figure 6A**). The results revealed three putative genes: CDK6, GTPBR10, and ZFHX4. It is well known that miRNA binds to the 3′-untranslated region of target genes to regulate expression. The expression of potential target genes were detected by qRT-PCR analysis of cells transfected with the miR-1263 mimics or inhibitor (**Figures 6B, C**). The results showed that CDK6 was the most probable target gene. To further confirm the interaction between miR-1263 and CDK6, luciferase reporter plasmids containing the WT or MUT CDK6 sequence were constructed (**Figure 6D**). The activity of the luciferase reporter was decreased when co-transfected with miR-1263 mimics as compared with the NC (**Figures 6E, F**). Moreover, the western blot results showed that inhibition of miR-1263 actually elevated CDK6 protein levels in 97H cells, while overexpression had an opposite effect (**Figure 6G**). These findings confirmed that miR-1263 directly targeted CDK6. Moreover, the results of the rescue experiments indicated that CDK6 expression was indirectly regulated by circERBIN *via* the circERBIN/miR-1263/CDK6 regulatory axis at the RNA and protein levels (**Figures 6H–K**). To explore the roles of CDK6 on HCC phenotypes, further analysis found that CDK6 mRNA expression was obviously upregulated in HCC tissues (**Figure 6L**). In addition, CDK6 expression was negatively correlated with miR-1263 in HCC tissues (**Figure 6M**).

### circERBIN Promotes HCC Progression *via* the miR-1263/CDK6 Axis

**Figure 7:**
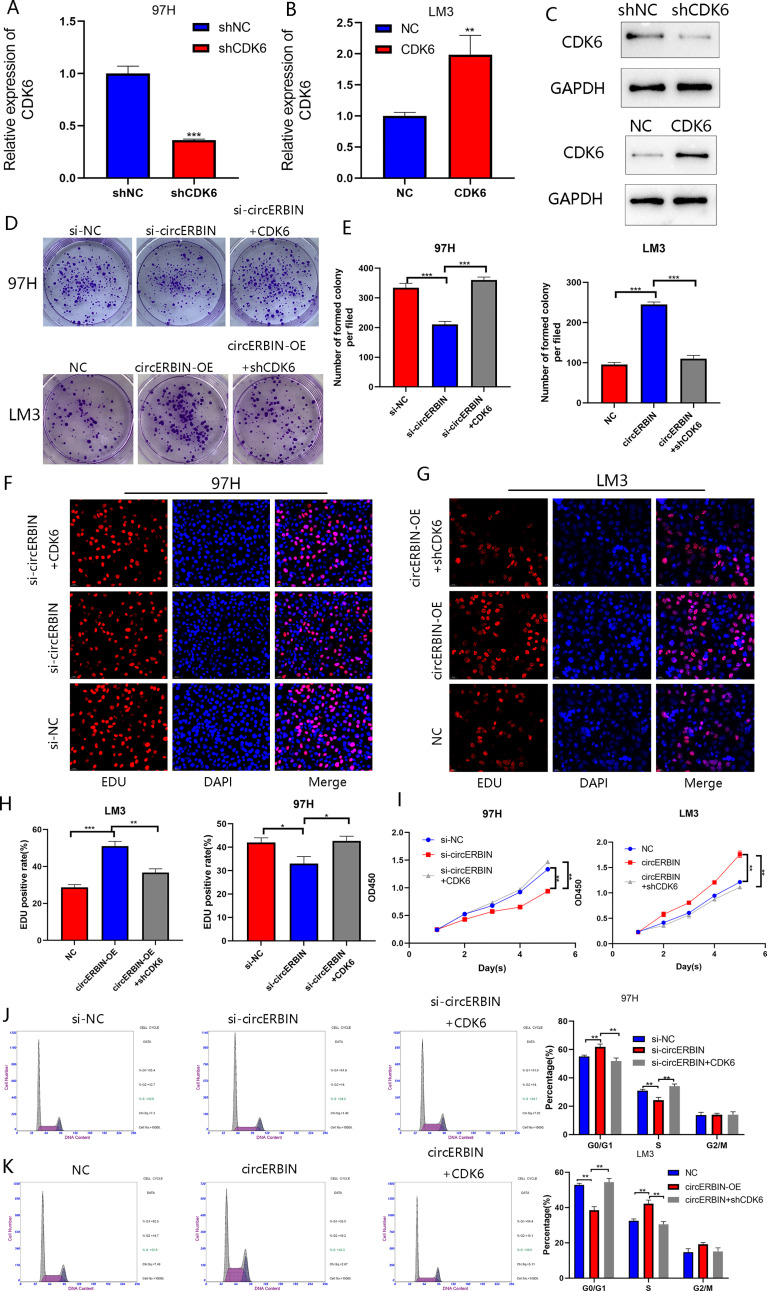
CircERBIN promotes the progression of HCC *via* the miR-1263/CDK6 axis. **(A–C)** Relative expression of CDK6 in 97H and LM3 cells transfected with shCDK6, CDK6, or NC as detected by qRT-PCR and western blot analyses. **(D–I)** The effects of circERBIN and CDK6 on HCC cells proliferation in HCC cell lines were evaluated by colony formation, EdU cell proliferation, and CCK-8 assays. **(J, K)** Flow cytometry showing that circERBIN and CDK6 affect G1 to S phase transition. (*p < 0.05, **p < 0.01, ***p < 0.001).

The efficiency of shCDK6 transfection was verified by qRT-PCR (**Figures 7A, B**) and western blot (**Figure 7C**) analyses. Transfection with shCDK6 significantly decreased CDK6 expression as compared with the shNC. Silencing of circERBIN downregulated the proliferation of 97H cells, while the addition of CDK6 reversed the inhibiting effects on cell growth as determined by the CCK-8 (**Figure 7I**), colony formation (**Figures 7D, E**), and EdU cell proliferation (**Figures 7F–H**) assays. In contrast, circERBIN-mediated growth was counteracted by transfection with shCDK6. Moreover, the addition of CDK6 reversed G1 arrest induced by silencing of circERBIN (**Figure 7J**). ShCDK6 also inhibited G1 to S phase transition accelerated by circERBIN overexpression (**Figure 7K**). IHC staining results for CDK6 in subcutaneous tumors in a previously described *in vivo* model confirmed the correlation between circERBIN and CDK6 (**Figures 3E, G**). In short, circERBIN promoted the progression of HCC *via* CDK6.

## Discussion

Mounting evidence has demonstrated that dysregulation of circRNAs plays critical roles in HCC tumorigenesis and progression through regulating key tumor regulatory genes. In view of the great importance of circRNAs in HCC, we previously revealed that circ_0011385 enhances HCC cell proliferation and tumor activity *via* regulation through the miR-361-3p/STC2 axis and that circCRIM1 facilitates HCC progression and angiogenesis by sponging miR-378a-3p (9, 11). Owning to the circular structures, circRNAs are more stable than the corresponding linear forms and are expected to be exciting targets for tumor drug discovery (12). Importantly, dysregulation of circRNAs has been extensively profiled, although the exact functions and molecular mechanisms of specific circRNAs in HCC progression remain to be fully elucidated.

The results of this study clarified that circERBIN originates from exons 2, 3, and 4 of the Erbin gene which plays critical roles in multiple malignancies, including lung adenocarcinoma, colorectal cancer, kidney renal papillary cell carcinoma, and particularly HCC (13–15). Erbin is reportedly elevated in HCC and promotes tumorigenesis by enhancing the ubiquitination and degradation of estrogen receptor-alpha (16). A recent study reported that circERBIN is a potential biomarker of the progression of colorectal cancer (10). Nevertheless, the expression pattern and function of circERBIN in HCC progression have yet to be determined. Hence, the present investigation focused on the role and potential mechanism of circERBIN in HCC.

The results of the present study showed that circERBIN is upregulated in HCC, which was consistent with the expression pattern of the linear form of Erbin. Additionally, higher circERBIN expression was associated with poorer clinicopathological features and prognosis. Furthermore, circERBIN facilitated the proliferation of HCC cells both *in vitro* and *in vivo via* regulation of the cell cycle. Based on the competing endogenous RNA mechanisms, we further assumed that circERBIN worked as a miRNA sponge in the cytoplasm. Bioinformatics, the RNA pull-down assay, and luciferase reporter gene assay were employed to predict and confirm that circERBIN directly targeted miR-1263. Then, we illustrated the tumor suppressive role of miR-1263 in HCC. Our data showed that miR-1263 was downregulated in the cytoplasm and inhibited the growth of HCC cells by targeting CDK6, which is indispensable for cell cycle progression to the G1 phase and G1/S transition (17). However, further investigations are needed to determine whether the effects of circ-ERBIN are dependent on the host Erbin gene.

Dysregulation of cell cycle proteins is a common characteristic of cancer cells, thus targeting cell cycle pathways has attracted considerable interest as a promising strategy for cancer therapy (18). As the key driver of cell division and G1/S transition, expression of CDK6 is tightly controlled in cancer cells. CDK6 expression is reportedly regulated by transcription factors, miRNAs, and long non-coding RNAs (19–21). Recent studies have reported that CDK6 is regulated by circRNAs and participates in circRNAs-mediated G1/S transition (22, 23). In this study, circERBIN upregulated CDK6 expression by sponging miR-1263. Furthermore, circERBIN-induced growth of HCC cells and cell cycle transition were abolished by CDK6 knockdown. To date, the United States Food and Drug Administration has approved several CDK6 inhibitors for the treatment of breast cancer and other malignancies (17, 24). The results of the present study outline a promising strategy for the use of CDK6 for treatment of HCC patients with positive circERBIN expression.

In conclusion, circERIN was upregulated in HCC tissues and cells and strongly associated with unfavorable clinicopathological features and prognosis. Furthermore, circERBIN can modulate the cell cycle to facilitate HCC progression *via* the miR-1263/CDK6 axis. Hence, blocking circ-ERBIN presents a potential therapeutic target to halt the progression of HCC.

## Data Availability Statement

The raw data supporting the conclusion of this article will be made available by the authors without undue reservation.

## Ethics Statement

The study protocols involving human participants were reviewed and approved by the Ethics Committee of the First Affiliated Hospital of Nanjing Medical University. The patients/participants provided written informed consent to participate in this study.

## Author Contributions

SY and YJ conducted the experiments and wrote this manuscript. FY, YS, and HL conducted parts of the experiments. SY, YJ, and YG analyzed the data. FZ, XW, and CZ conceived and supervised this study. All authors contributed to the article and approved the submitted version.

## Funding

This work was financially supported by the National Natural Science Foundation of China (grant nos. 81870488, 81530048, and 31930020).

## Conflict of Interest

The authors declare that the research was conducted in the absence of any commercial or financial relationships that could be construed as a potential conflict of interest.

## Publisher’s Note

All claims expressed in this article are solely those of the authors and do not necessarily represent those of their affiliated organizations, or those of the publisher, the editors and the reviewers. Any product that may be evaluated in this article, or claim that may be made by its manufacturer, is not guaranteed or endorsed by the publisher.
